# Autologous Thymic Organoids Support Functional T-cell Education and Enhance Antitumor Immunity in Humanized Mice with Melanoma Xenografts

**DOI:** 10.1158/2767-9764.CRC-25-0357

**Published:** 2025-11-24

**Authors:** J. Jason Morton, Stephan A. Ramos, Nathaniel Alzofon, Stephen B. Keysar, Lucas H. Armitage, Alexander S. Baker, Jessie M. Barra, Tugs-Saikhan Chimed, Phuong N. Le, Cera Nieto, Alice N. Weaver, Carissa M. Thomas, Bettina Miller, William Robinson, Theresa M. Medina, Dexiang Gao, Holger A. Russ, Antonio Jimeno

**Affiliations:** 1 Division of Medical Oncology, Department of Medicine, University of Colorado School of Medicine, Aurora, Colorado.; 2 Charles C. Gates Center for Regenerative Medicine, University of Colorado School of Medicine, Aurora, Colorado.; 3 Diabetes Institute, University of Florida, Gainesville, Florida.; 4 Department of Pharmacology and Therapeutics, University of Florida, Gainesville, Florida.; 5 Department of Otolaryngology, University of Colorado School of Medicine, Aurora, Colorado.; 6 Department of Pediatrics, University of Colorado School of Medicine, Aurora, Colorado.; 7 Department of Biostatistics and Informatics, Colorado School of Public Health, University of Colorado Anschutz Medical Campus, Aurora, Colorado.

## Abstract

A fundamental limitation of *HLA-A* matched humanized mice (mHM), generated with donor hematopoietic stem and progenitor cells and bearing patient-derived xenografts, is that the fidelity of the interaction between the immune system and implanted tumors is limited by lack of thymic T-cell education. We created a model with patient-matched thymic and tumor tissue by using patient peripheral blood mononuclear cells reprogrammed into induced pluripotent stem cells to generate stem cell–derived thymic organoids (sTOs). When implanted in thymectomized mHM, which were subsequently engrafted with patient melanoma tissue (autologous, or mHM_TA_), sTOs expressed thymic tissue markers EPCAM, KRT5, and KRT8 and contained developing double-positive T cells. mHM_TA_ had more activated HLA-DR+ T cells in their spleens. Tumor growth in mHM_TA_ was significantly slower compared with mHM, and both tumor volume and viable melanoma content were significantly decreased in mHM_TA_. This was associated with an increase of intratumoral activated T cells in mHM_TA_. Whole-exome sequencing followed by intratumoral heterogeneity analysis identified seven candidate neoantigens that were selected against in the mHM_TA_ model, suggesting more effective identification and T cell–driven tumor clearance. This study shows the feasibility of generating patient-specific, thymus-bearing mice and provides a promising new tool in immunology and immunotherapy development.

**Significance:**

A humanized mouse xenograft model, in which T cells are educated in a patient-derived implanted thymic organoid autologous to the tumor, provides a more faithful and complete environment to study cancer immunity.

## Introduction

Immunotherapy development has been hindered by the lack of available preclinical systems to accurately model tumor–stroma interactions. Humanized mouse (HM) models have shown potential to fill this need (1, 2). Human hematopoietic stem and progenitor cells (HSPC), derived from either donated umbilical cord blood (UCB) or the peripheral blood of adults treated with filgrastim, colonize mouse bone marrow and give rise to B cells, monocytes, and T-cell lineages. Although myeloid lineages do not require developmental stimuli beyond those present in the bone marrow, progenitor T (proT) cells must migrate to the thymus in which they undergo T-cell lineage development and T-cell receptor (TCR) recombination, followed by positive and negative selection to produce mature T cells. In HM, CD34^+^CD7^+^CD5^+^ proT cells traffic to the murine thymus to mature before emigrating into the periphery (3). In some cases, these T cells can be activated by immune checkpoint inhibitors and specifically attack tumor cells, but such activity seems stochastic (4, 5). One possible reason for unpredictable responses in HM bearing patient-derived xenografts (PDX) is that the TCR repertoire of the human T cells educated by a murine thymus is dissimilar to that produced by a human thymus (6). Prior studies have shown that HM T cells are often anergic and incapable of responding properly to foreign antigens or tumor-associated neoantigens (neoAg; refs. 7, 8).

In response to this obstacle, fetal thymic tissue was implanted into HM models to produce a more representative T-cell repertoire (9, 10). Although this and other similar strategies using neonatal thymic tissue and HSPCs can be effective, thymic tissue is not readily available, and even when used, the resultant T cells have been educated in a thymic environment allogeneic to the tumor (11). In prior work, we developed a direct differentiation protocol to generate thymic epithelial progenitor cells (TEP) from multiple induced pluripotent stem cell (iPSC) lines (12, 13). Upon transplantation into athymic nude mice, TEPs further differentiated into functional thymic epithelial cells (TEC) with the ability to educate mouse T cells (12). Additionally, although differentiation of TEPs from human iPSCs has been demonstrated by us and others, we further developed this approach to generate functional TECs *in vitro* within stem cell–derived thymic organoids (sTO; ref. 13). The generation of patient-autologous thymic tissue via iPSC technology would enable personalized immunotherapy and accurate identification of neoAgs for cancer vaccine development.

To this end, we used peripheral blood mononuclear cells (PBMC) from a patient with metastatic melanoma to generate iPSCs and combined iPSC-derived TEPs, HSPCs, and mesenchymal cells to generate functional isogenic sTOs. These were then implanted under the kidney capsule of NOD.Cg-*Prkdc*^*scid*^*Il2rg*^*tm1Wjl*^*/*SzJ (NSG) mice previously humanized with cord blood *HLA-A* matched to that of the patient PBMCs, followed by implantation of the patient’s melanoma tissue. To ensure that the T cells were not trafficked to the murine thymus, we surgically thymectomized the mice prior to humanization. Using this system, we report that sTO-educated T cells are responsive to antigenic stimulation, delay autologous PDX tumor growth, and cause genomic changes in autologous PDX tissue consistent with immunoediting.

## Materials and Methods

### Human and animal regulatory approvals

Human subject studies were approved by the Colorado Multiple Institutional Review Board (COMIRB-14-0842) in accordance with the Declaration of Helsinki, International Ethical Guidelines for Biomedical Research Involving Human Subjects, Belmont Report, or U.S. Common Rule. Written informed consent was obtained from the single patient (CUHM009) whose blood and tumor tissue were used for this pilot study. Animal procedures were approved by the University of Colorado Institutional Animal Care and Use Committee (IACUC-00202). PDX generation and animal care have been previously reported (14).

### sTO generation

The collection and isolation of patient HSPCs and PBMCs have been previously described (2). Induction of iPSCs and subsequent differentiation to TEPs were conducted as previously described (13). At approximately day 18 to 31, TEPs were harvested using a 1-mL pipette, mechanically dissociated into small clumps, and transferred to a 1.5-mL tube.

For initial sTO characterization, allogeneic HSPCs were isolated by magnetic-activated cell sorting (Miltenyi Biotec; cat. #130-100-453) from UCB units previously obtained from the University of Colorado cord blood bank (http://www.clinimmune.com). Differentiation day 18 to 25 TEPs were mechanically dissociated into small clumps and combined with isolated allogeneic HSPCs at a 1:20 HSPC:TEP ratio (2e5 HSPCs:1/2 × 24-well TEPs) in a 1.5-mL tube. Cells were pelleted in a swinging-bucket centrifuge at 1,200 RPM for 3 minutes and resuspended in 5 μL/sTO of RB27 medium supplemented with SCF (100 ng/mL), VEGF (50 ng/mL), FGF2 (10 ng/mL), IL7 (20 ng/mL), and FLT3-L (10 ng/mL; Thermo Fisher Scientific). SM1 without vitamin A (STEMCELL Technologies, cat. #05731) was substituted for B27 (15). Immediately before use, 2-phospho-L-ascorbic acid trisodium salt (Sigma-Aldrich, cat. #49752) was added at 50 μg/mL. A 5 μL drop was placed on a 0.4-μm Millicell Transwell insert (MilliporeSigma, cat. #PICMORG50) with two to three sTOs per membrane ∼1 cm apart. Transwells were placed in six-well plates containing 1.5 mL of RB27 media. Media were changed every 2 to 3 days. For their initial characterization, sTOs were cultured in this manner for up to 6 weeks before harvest. As it was observed that the expression of key thymic markers began to decline after 4 weeks, those used in *in vivo* studies were cultured a maximum of 4 weeks prior to implantation.

For *in vivo* experiments, the same *HLA-A*–matched HSPCs employed to humanize the mice were combined with previously karyotyped normal CUHM009 patient-derived TEPs at an approximate 1:20 ratio. The mixture was centrifuged at 1,200 RPM for 3 minutes, the supernatant was removed, and 15 μL aliquots were implanted into the kidney capsules of the HM. Representative sTOs were harvested 10 to 15 weeks after implantation for analysis.

### sTO characterization

For *in vitro* experiments, flow cytometry was performed on a Cytek Aurora flow cytometer (Cytek, RRID: SCR_019826) to characterize (i) proT cells using CD34, CD7, and CD5 antibodies (BioLegend, cat. #343608, RRID: AB_2228972; cat. #343114, RRID: AB_2563941; and cat. #364009, RRID: AB_2564505) at 1:20, 1:60, and 1:40, respectively, and (ii) T cells using CD3, CD4, CD8, CD45, and TCRα/β antibodies (BioLegend, cat. #344806, RRID: AB_10549300; cat. #300520, RRID: AB_389333; cat. #300916, RRID: AB_756152; cat. #304025, RRID: AB_893341; and cat. #306720, RRID: AB_10639947) at 1:100, 1:100, 1:100, 1:180, and 1:60, respectively. For qPCR analysis, reverse transcription of RNA was performed using an iScript cDNA Synthesis Kit (Bio-Rad Laboratories, cat. #1708891BUN). For immunofluorescence (IF), 4- to 10-μm tissue sections were cut from paraffin or optimal cutting temperature–embedded tissue blocks. Deparaffination and antigen retrieval have been previously described (12). Slides were stained using the following antibodies and dilutions: CD3 (Abcam, cat. #ab5690, RRID: AB_305055) 1:100, CD4 (Santa Cruz Biotechnology, cat. #sc-19641, RRID: AB_627055) 1:100, CD8 (Abcam, cat. #ab60076, RRID: AB_940921) 1:100, EPCAM (BioLegend, cat. #324202, RRID: AB_756076) 1:200, KRT5 (Abcam, cat. #ab52635, RRID: AB_869890) 1:100, KRT8 (Santa Cruz Biotechnology, cat. #sc-8020, RRID: AB_627857) 1:100, panKRT (Abcam, cat. #ab9377, RRID: AB_307222) 1:300, and PDGFRα (Abcam, cat. #ab203491, RRID: AB_2892065) 1:500. The Z-stack or snap images were taken with a Zeiss LSM 800 microscope (Carl Zeiss AG).

### HSPC collection, expansion, and engraftment in mice and renal capsule sTO implantation

HSPC purification, expansion, and engraftment were conducted as previously described (4). Three independent HM cohorts were generated for this study using HSPCs derived from donor UCB procured from Clinimmune (www.clinimmune.com) or STEMCELL Technologies (cat. #70007). Three separate experiments were conducted using independent HLA-A matched HM (mHM)/HLA-A matched thymectomized patient-autologous sTO (mHM_TA_) cohorts from different cords. High-resolution HLA typing matched the patient (HLA-A*02:01/*24:01) with UCB from three donors with one matching *HLA-A* allele (UCB120, *02:01/*25:01; UCB122, *02:01/*68:01; and UCB139, *02:01/*11:01; Supplementary Table S1; ref. 2). HSPCs were isolated after 8 to 9 days of *in vitro* expansion. The UCB120, UCB122, and UCB139 cultures had 70.2%, 90.5%, and 60.7% CD34^+^ HSPCs, respectively (Supplementary Fig. S1). One week after humanization, sTOs were implanted in HM and NSG controls. Animals were anesthetized with isoflurane. A 15-μL aliquot of sTO cell slurry (equaling material from approximately six sTOs mixed with 1,200,000 HSPCs from those expanded to humanize the mice) was injected under the kidney capsule, as described previously (16).

### FACS and flow cytometry

Tumor and mouse tissues were prepared for cytometric analysis as previously described (4). Cell sorting was performed using a MoFlo XDP70 (Beckman Coulter), an Astrios EQ (Beckman Coulter, RRID: SCR_018893 and RRID: SCR_019648), or a FASCDiscover S8 spectral sorter (BD Biosciences, RRID: SCR_026674), and flow cytometry was completed on a ZE5 (Bio-Rad Laboratories, RRID: SCR_019712) or an Aurora Spectral Analyzer (Cytek, RRID: SCR_019826) and analyzed using FlowJo software (Becton Dickinson; RRID: SCR_008520). Human blood cells were identified by CD3, CD4, CD8, PD-1, HLA-DR (BioLegend, cat. #300448, RRID: AB_2563468; cat. #300537, RRID: AB_2562051/300508 and RRID: AB_314076; cat. #301050, RRID: AB_2562055/301049 and RRID: AB_2562054; cat. #367422, RRID: AB_2721517/329952 and RRID: AB_2566364; and cat. #327018, RRID: AB_2566389/307616 and RRID: AB_493588), CD45RA, and CD45RO (Becton Dickinson, cat. #612846, RRID: AB_2870168; cat. #562791, RRID: AB_3713569 and BioLegend, cat. #304128, RRID: AB_10708880; cat. #304208, RRID: AB_314424) antibodies, all at 1:10. Mouse CD45 (BioLegend, cat. #103116, RRID: AB_312981) antibody was used to exclude murine leukocytes.

### IHC and multiplexed ion beam imaging

IHC analyses were performed as described (1). Samples were submitted to the Human Immune Monitoring Shared Resource at the University of Colorado School of Medicine for multiplexed ion beam imaging (MIBI) analysis using their validated antibody panel. Stained slides were analyzed using MIBItracker software (Ionpath, RRID: SCR_023605).

### Whole-exome sequencing

Genomic DNA samples were submitted to the Genomics Shared Resource at the University of Colorado Cancer Center. Following quantitation using a Qubit Fluorometer (Thermo Fisher Scientific), 200 ng of genomic DNA from each sample was used for library preparation following the manufacturer’s protocol. For whole-exome sequencing library preparation, the SureSelect Human All Exon V6 r2 (Agilent Technologies, cat. #S07604514) target enrichment kit was used with the Agilent SureSelectXT system. Libraries were sequenced on an Illumina NovaSeq 6000 Instrument, RRID: SCR_016387, using an S4 flow cell with v1.5 chemistry.

### Next-generation sequencing data analysis and bioinformatics

A pipeline was constructed modeled from the GATK (RRID: SCR_001876) Best Practices for somatic variant calling (17). Adapter sequences were trimmed using the Agilent Genomics NextGen Toolkit (AGeNT) trim module. Reads were then aligned to GRCh38.p13 (RefSeq assembly accession: GCF_000001405.39) using BWA (RRID: SCR_010910; ref. 18) as implemented in the BWA kit (github.com/lh3/bwa/tree/master/bwakit). Mutect2 was used in multi-sample mode to call all somatic variants from all tumors simultaneously, thereby increasing the sensitivity and specificity of variant calls. Further methods are discussed in the Supplementary Methods.

### Statistics

Continuous measurements, such as biomarkers, antigens, or ligands via *in vitro* and *in vivo* (using ≥5 mice/group) experiments, were compared using Brown–Forsythe ANOVAs with Tukey multiple comparison, Wilcoxon rank-sum tests, or a two-sided *t* test, as appropriate. Linear mixed-effects models were used to model tumor volume and tumor burden, with the tumor burden defined as the total volume of all tumors on the mouse. Kaplan–Meier plotting was used to display the progression-free survival (PFS), and a log-rank test was used to compare the PFS between groups. Binomial regression to calculate an OR, and an ANOVA using an overall *F* test, followed by FDR adjustment for multiple tests was used to estimate variant allele frequency. Calculations were done using GraphPad Prism version 8.3 and the R software. Data are represented graphically as mean ± SEM throughout. For all tests, significant *P* values are conveyed as follows: *, *P* ≤ 0.05; **, *P* ≤ 0.01; ***, *P* ≤ 0.001; ****, *P* ≤ 0.0001.

## Results

### *In vitro* development and differentiation of sTOs give rise to T cells

**Figure 1.**
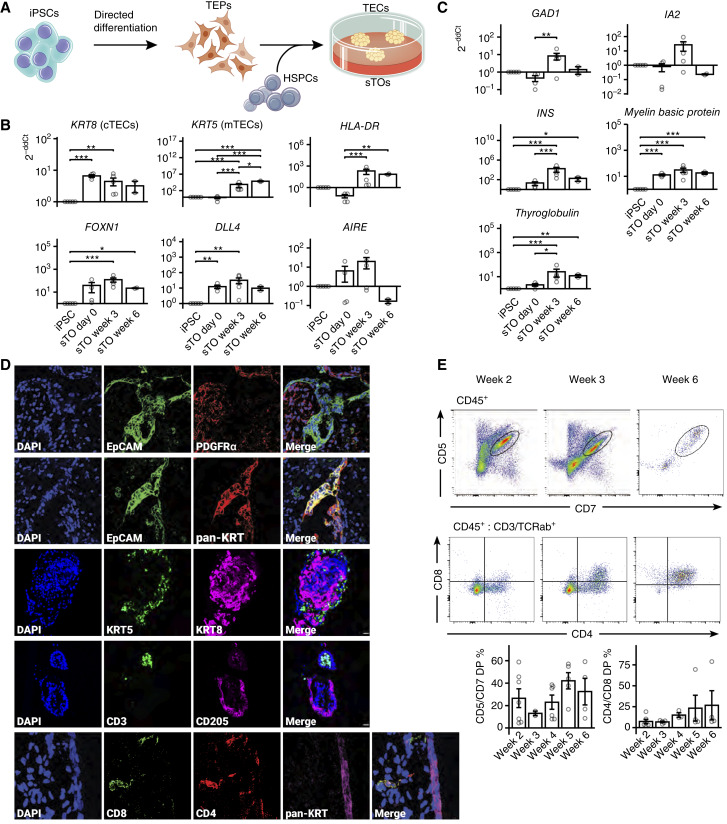
*In vitro* patient-specific sTO characterization. **A,** Differentiation of patient-derived iPSCs to TEPs and subsequent production of sTOs. iPSCs are differentiated into TEPs. Coculture of allogeneic (for *in vitro* examination of the sTOs) or *HLA-A*–matched HSPCs (for *in vivo* experiments) at the air–liquid interface produces sTOs with functional TECs. **B,** qPCR examination of key thymic marker expression in sTOs *in vitro*. The fold change (2^−ddCt^) in the expression of these genes in sTO cultures at 0-, 3-, and 6-week timepoints was compared with that of undifferentiated iPSCs. Significance was determined using a one-way ANOVA with a Tukey multiple comparison test. cTEC, cortical TEC; mTEC, medullary TEC. **C,** qPCR examination of tissue-restricted antigen expression *in vitro*, indicative of AIRE activity, calculated and with significance as determined above. **D,** IF microscopy of sTOs after 3 weeks of culture shows cell structures expressing thymic markers and identifies presumptive T cells within these structures. DP T cells are indicated by white arrows. Magnification is 40×. Scale bar, 100 μm. **E,** Cytometry of dissociated sTOs to identify maturing T cells. CD5/7 proT cells were identified after gating CD45^+^ cells, whereas CD4/8^+^ T cells were identified after gating CD45 and CD3/TCRαβ cells. *P* values: *, ≤0.05; **, ≤0.01; *** ≤0.001.

Building on studies in which we implanted TEPs generated from cord blood–derived iPSCs in the renal capsule to stimulate the development of murine T cells in nude mice (12) and our recent demonstration of sTO cultures *in vitro* (13), we first generated iPSCs from the PBMCs of a patient with melanoma CUHM009 by episomal reprogramming (Supplementary Fig. S2A and S2B; refs. 12, 19). We then used iPSCs to generate TEPs via direct differentiation, as described (12, 13), and established sTOs by aggregating CUHM009 patient iPSC-derived TEPs with allogenic, human cord blood–derived CD34^+^ HSPCs at the air–liquid interface (Fig. 1A). Subsequent qPCR analysis of sTOs showed the upregulation of key thymic markers keratin 8 (*KRT8*), keratin 5 (*KRT5*), major histocompatibility complex, class II, DR alpha (*HLA-DRA*), Forkhead Box N1 (*FOXN1*), and delta like canonical Notch ligand 4 (*DLL4*) after 3 weeks and/or 6 weeks, indicating the maturation of iPSC-derived TEPs to TECs *in vitro* (Fig. 1B). We also observed increased expression of the autoimmune regulator (*AIRE*) transcript in sTOs after 3 weeks in culture, not previously observed *in vivo* using the xenogeneic nude model (12). Further qPCR analysis identified expression of tissue-restricted antigens *GAD1*, *IA2*, *INS*, myelin basic protein (*MBP*), and thyroglobulin (*TG*) in the sTOs at 3 weeks, representing different germ layers and corroborating functional *AIRE* expression (Fig. 1C).

IF analysis of sTO sections for epithelial and mesenchymal markers, EPCAM and PDGFRα, respectively, revealed presumptive thymic epithelial structures surrounded by mesenchymal cells [Fig. 1D (panel 1)]. Co-staining of EPCAM+ cells with a pan-keratin antibody showed co-expression in almost all epithelial cells [Fig. 1D (panel 2)]. To identify medullary and cortical TECs, sections were stained for KRT5 and 8, respectively. Some KRT5/8 double-positive (DP) cells, indicative of developing TECs [Fig. 1D (panel 3)], were present. Additionally, CD3^+^ lymphocytes and CD205^+^ TECs were present in sTOs, indicating the development of distinct epithelial and lymphocyte compartments in them [Fig. 1D (panel 4)]. Further IF analysis of sTO sections for T-cell markers CD4 and CD8 showed DP developing T cells located near pan-KRT–positive thymic structures [Fig. 1D (panel 5)].

We quantified the emergence of proT cells, marked by CD5 and CD7, by flow cytometry and found low levels of proT cells at week 2 followed by a substantial increase at week 3 and throughout the culture period assayed [Fig. 1E (panel 1)]. Quantitative flow analysis of CD4 and CD8 revealed increasing numbers of developing DP T cells starting at week 2, which were not present in isolated CD34^+^ HSPCs at the start of sTO culture [Fig. 1E (panel 2)]. This analysis also identified large populations of single positive CD4 and CD8 T cells at weeks 2 and 3.

### Development of a thymectomized HM model

**Figure 2.**
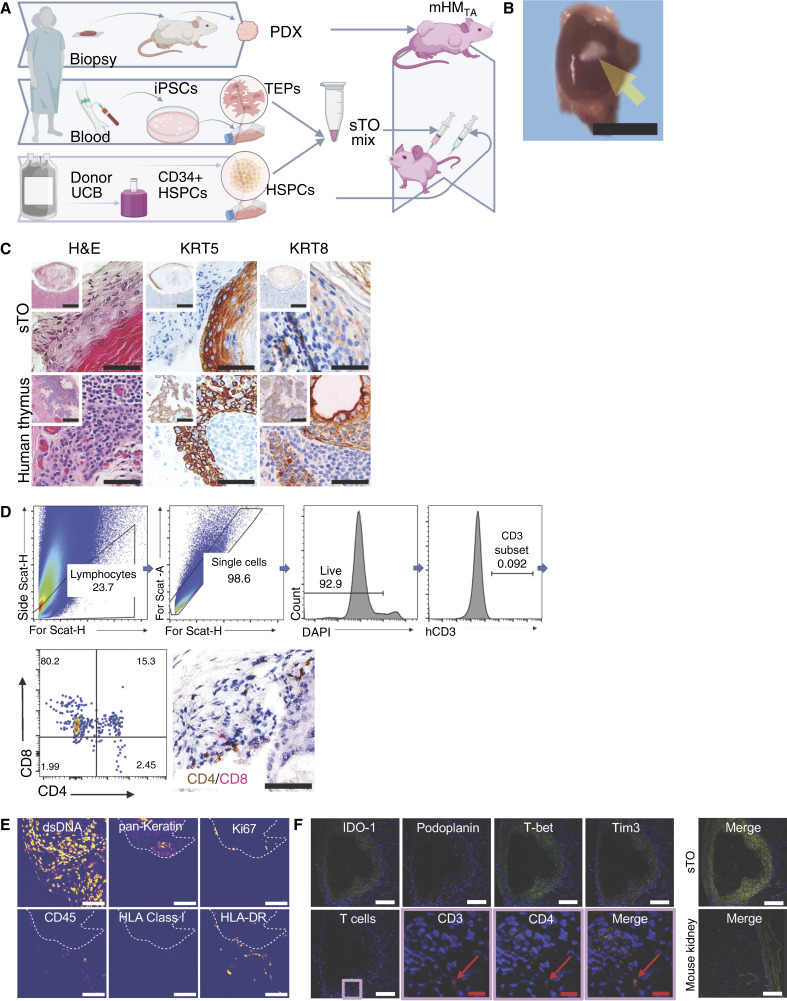
*In vivo* sTO characterization. **A,** Overview of mHM_TA_ production. Tumor biopsies are implanted as PDXs on NSG mice to expand prior to use in the HM. Patient blood is also drawn at the time of biopsy and reprogrammed into iPSCs and subsequent sTOs, using *HLA-A*–matched cord blood–derived HSPCs. The sTOs are surgically implanted into the kidney capsule of thymectomized mHM produced with the same HSPCs used to produce the mHM_TA_. After human peripheral blood cells have been identified in the HM, PDX tissue is implanted into the mHM and mHM_TA_. **B,** Picture of a successfully engrafted sTO growing under the kidney capsule, indicated by the yellow arrow. Scale bar, 1 mm. **C,** Top row (inset): IHC comparison of human thymic tissue with engrafted sTO tissue shows that the sTO tissue expresses both human KRT5 and KRT8. Magnification is 10×; scale bar, 100 ×m. Middle row: Magnified portion of the boxed region in the above sTOs. Columns 1–2, magnification is 20×; scale bar, 50 ×m. Column 3, magnification is 40×; scale bar, 25 μm. Bottom row: Human thymus, for comparison. Magnification is 20×; scale bar, 50 μm. H&E, hematoxylin and eosin. **D,** Cytometry with all gating of a successfully engrafted sTO shows that the tissue harbors CD4/8 DP T cells undergoing thymic selection and maturation. IHC dual staining of engrafted sTO tissue shows that the tissue harbors CD4/8 DP T cells undergoing thymic selection and maturation. Magnification = 40×; scale bar, 25 μm). Cytometry shows that the tissue harbors CD4/8 DP T cells undergoing thymic selection and maturation. **E,** MIBI images of an engrafted sTO show that the cellular tissue expresses pan-keratin and ki67, markers of viability. The presence of human CD45^+^ cells, as well as the expression of HLA class I and HLA-DR proteins, shows its role in immune cell education. **F,** MIBI images of the sTO also show thymus-specific protein expression. Top row: IDO-1, podoplanin, T-bet, and Tim3. Their merged expression is shown in the final image. Bottom row: Images of sTO-associated T cells, initially showing an overview of the sTO and subsequently an example of a CD3/4+ T cell enlarged in images. The final image is of a nongrafted mouse kidney for comparison of all markers. White scale bar, 100 μm. Red scale bar, 25 μm.

Although HM develop human T cells, these undergo positive/negative selection against murine antigens in the murine thymus, and to ensure that an engrafted sTO was the major site of T-cell education, we established and validated a surgical protocol to thymectomize NSG mice. We then generated mice to test the capacity of sTOs to educate T cells *in vivo*, by comparing PDX tumor growth on (i) *HLA-A*–matched HM in which T cells were educated in the murine thymus (mHM), (ii) thymectomized mHM in which T cells were educated in patient-autologous sTOs implanted under the kidney capsule (mHM_TA_), and (iii) non-humanized NSG controls (Fig. 2A).

### sTO engraftment yields human thymic tissue *in vivo*

We were able to identify multiple mice from each repeat study cohort with sTOs in the renal capsule (Fig. 2B), as assessed through human KRT5 and KRT8 staining by IHC (Fig. 2C; Supplementary Fig. S3). In contrast, murine thymus tissue removed from mHM expressed only murine cytokeratins, although human KRT8 was minimally cross-reactive to murine thymic tissue. The murine thymic tissue also contained human T cells and maturing DP T cells (Supplementary Fig. S4). We measured CD4/CD8 DP lymphocytes in the sTOs by flow cytometry and IHC double staining (Fig. 2D). Further characterization of the sTOs was done with MIBI using positive pan-keratin staining to identify the sTO (Fig. 2E). Several previously reported thymus-associated proteins expressed within the sTO were identified; IDO-1 and podoplanin are expressed in the thymic medulla (20, 21), T-bet is a transcription factor linked to the olfactory bulb and thymus during embryogenesis (22), and TIM3 has been associated with dendritic cells, along with other cells within the thymus [Fig. 2F (top); ref. 23]. We also identified a population of T cells within and around the sTOs and characterized CD4^+^ T cells within these structures [Fig. 2F (bottom)]. The expression of these markers and T cells were absent in nongrafted mouse kidney examined as a comparison.

### mHM_TA_ produce physiologically relevant T-cell populations

**Figure 3.**
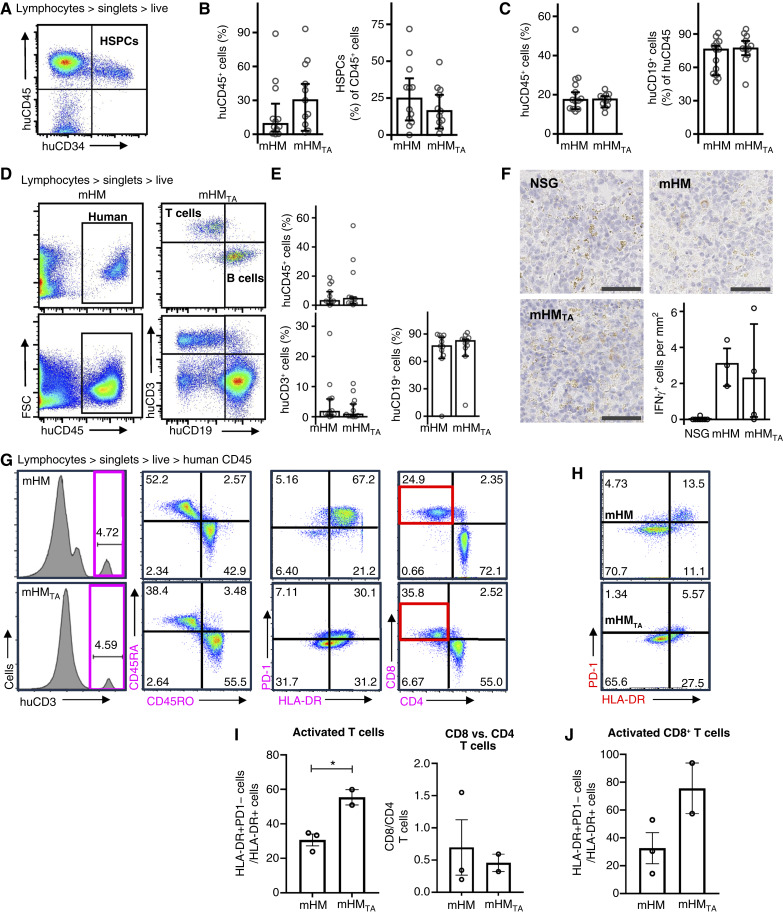
Examination of T-cell development and activity in the mHM_TA_. **A,** Representative cytometry of the human CD45^+^ cells and HSPCs present in mHM and mHM_TA_ bone marrow. **B,** Comparison of the average percentage of human CD45^+^ cells and CD34^+^ HSPCs within this CD45^+^ cell population within mHM and mHM_TA_ bone marrow (*P* = 0.118 and 0.379, respectively, Wilcoxon rank-sum test). **C,** Comparison of peripheral human CD45^+^ lymphocytes in mHM and mHM_TA_ (*P* = 0.728, Wilcoxon rank-sum test) and of CD19^+^ B cells in mHM and mHM_TA_ (0.224, Wilcoxon rank-sum test). **D,** Cytometric analysis and a comparison of human CD45^+^ cells, as well as T-cell and B-cell populations, found in the spleens of mHM and mHM_TA_. **E,** Comparison of the average human CD45^+^ cell populations, human T cells, and human B cells on mHM and mHMTA spleens (*P* = 0.844, 0.450, and 0.646, Wilcoxon rank-sum test, respectively). **F,** IFNγ expression was detected in the spleen of both mHM and mHM_TA_ (*P* < 0.005). **G,** Gating and representative cytometry of T cells in mHM and mHM_TA_ show relative populations used to analyze T-cell activation. Purple boxes in the first panels show the T-cell populations gated in the remaining scatter plots. The red boxes show the CD8^+^ T-cell populations gated for subsequent analysis. **H,** Representative cytometry of the CD8^+^ T cells, gated from the red boxes in the previous panel. **I,** Comparisons of the ratio of HLA-DR + PD1– T cells with all HLA-DR+ T cells shows an increasingly activated T-cell population in mHM_TA_ (*P* = 0.0207, two-group *t* test) and a nonsignificantly increased population of CD4^+^ T cells. **J,** Comparison of the HLA-DR + PD1– population with the total HLA-DR+ T-cell population among CD8^+^ T cells also reveals a trend toward increased activation in mHM_TA_. *P* values: *, ≤0.05.

We analyzed the blood and tumor growth from the mHM and mHM_TA_ by comparing only those mHM (*n* = 9) and mHM_TA_ (*n* = 10) for which peripheral blood consisted of at least 10% human cells (Supplementary Fig. S5). There were no significant differences in HSPC engraftment and subsequent humanization between mHM and mHM_TA_ groups (Fig. 3A and B). Neither the percentage of human CD45^+^ cells in mHM and mHM_TA_ blood was significantly different (*P* = 0.728, Wilcoxon rank-sum test; Fig. 3C) nor was the fraction of B cells among these lymphocytes (*P* = 0.224; Wilcoxon rank-sum test; Fig. 3C). As the *Prkdc* mutation in the NSG mouse strain prevents murine T-cell maturation, very few putative T cells were observed in NSG mice or in NSG mice in which sTOs had been implanted. When engrafted with human HSPCs, thymectomized mHM with no sTO implants also produced essentially no T cells (Supplementary Fig. S6A), although these mice harbored B-cell and monocyte populations similar to those observed in mHM controls (Supplementary Fig. S6B).

We next examined human lymphocyte populations in mHM and mHM_TA_ spleens (Fig. 3D). No differences existed in the percentage of CD45^+^ lymphocytes or in T-cell or B-cell populations between models (*P* = 0.844, 0.450, and 0.646, respectively, Wilcoxon rank-sum test; Fig. 3E). Although both models produced lymphocytes capable of producing IFNγ (Fig. 3F), there were more IFNγ-positive cells in mHM_TA_ versus mHM (1.13 vs. 0.93 cells per mm^2^ of tissue examined; Wilcoxon rank-sum test).

We characterized the sub-populations and activation status of T cells in mHM_TA_ and mHM spleen (Fig. 3G and H). Although the ratios of CD45RA to CD45RO cells were similar, a greater percentage of T cells were activated (PD-1–/HLA-DR+) in mHM_TA_, [*P* = 0.0207, two-group *t* test; Fig. 3I (first graph)] indicative of increased potential for long-term activation (24). A greater fraction of these T cells was also CD45RO/HLA-DR^+^ (Supplementary Fig. S7). Higher activation in mHM_TA_ was also documented when all mHM and mHM_TA_, with a sufficiently large splenic T-cell population analyzed (*P* = 0.0082, two-group *t* test; Supplementary Fig. S8). We observed a trend toward larger CD4^+^ T-cell populations in mHM_TA_ [Fig. 3I (second graph)], suggesting better immune reconstitution and T-cell education. Likewise, when CD8^+^ T cells, for which education and subsequent activity would be maximized in an *HLA-A*–matched model, were gated [Fig. 3G (red box)] and examined, their PD-1/HLA-DR+ population was also trended upward (Fig. 3H and J).

### Tumor growth dynamics change in mHM_TA_

**Figure 4.**
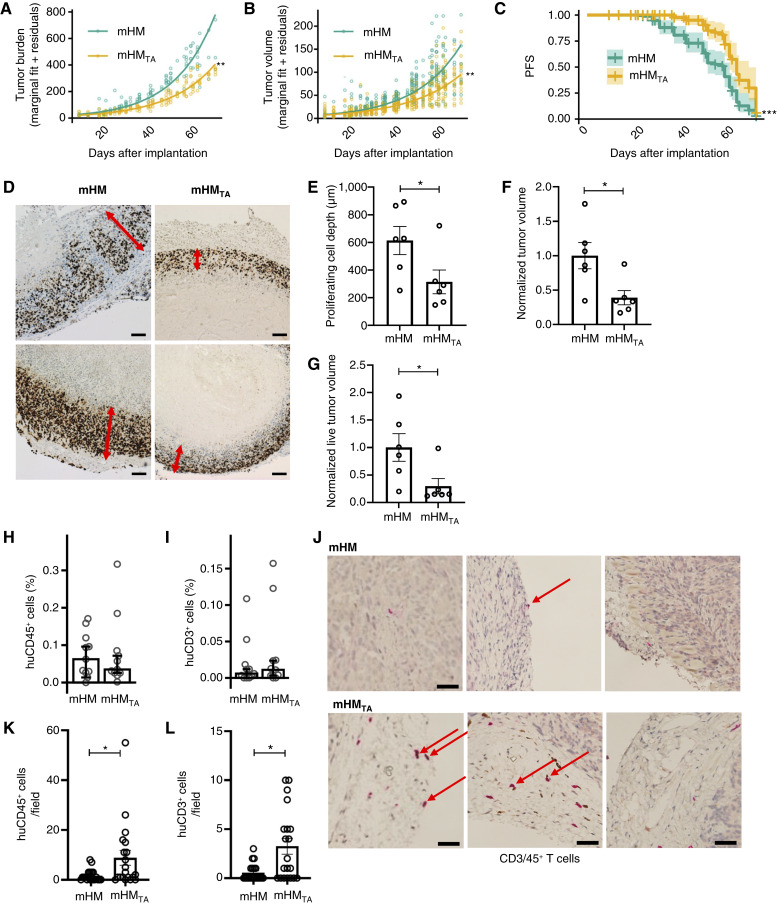
Tumor analysis in mHM and mHM_TA_. **A,** Comparison of the average mouse tumor burden on mHM (*n* = 27 on nine mice) and mHM_TA_ (*n* = 30 on 10 mice) shows that PDX growth on mHM_TA_ is significantly slowed (*P* = 0.007, linear mixed-effects model). **B,** Comparison of tumor volumes also indicates slower PDX growth in mHM_TA_ (*P* = 0.003, linear mixed-effects model). For **A** and **B**, the marginal fit plus the residuals are plotted for each mouse/tumor at each timepoint are shown as circles. **C,** PFS (defined as a 20% increase in volume from the starting date) analysis of mHM and mHM_TA_ tumors (*P* = 0.0006, cumulative HR). **D,** Ki67 IHC staining of mHM and mHMTA tumors showing the depth of thickness of living cells surrounding the necrotic tumor core. Magnification is 5×; scales bar, 100 μm. **E,** Quantification of the depth of these proliferating cells from the tumor edge (*P* = 0.048, two-group *t* test). **F,** Normalized comparison of mHM and mHM_TA_ tumor volumes reveals a significant reduction in mHM_TA_ (*P* = 0.019, two-group *t* test). **G,** Likewise, a normalized comparison of mHM and mHM_TA_ live tumor volumes indicates a significant reduction in mHM_TA_ (*P* = 0.034, two-group *t* test). **H,** A comparison of human CD45^+^ cell infiltration in the tumors of mHM and mHM_TA_ (*P* = 0.743, Wilcoxon rank-sum test). **I,** A comparison of T-cell infiltration in mHM and mHM_TA_ tumors (*P* = 0.468, Wilcoxon rank-sum test). **J,** IHC of CD3/45+ T cells (indicated by arrows) infiltrating the mHM and mHM_TA_ tumors. Magnification is 40×; scale bar, 50 μm. **K,** Quantification of human CD45^+^ lymphocytes within the tumor tissue and capsule indicates increased infiltration within mHM_TA_ tumors (*P* = 0.0207, Wilcoxon rank-sum test). **L,** Quantification of human CD3^+^ T cells within the tumor tissue and capsule likewise revealed increased infiltration in mHM_TA_ tumors (*P* = 0.0107, Wilcoxon rank-sum test). *P* values: *, ≤0.05; **, ≤0.01; ***, ≤0.001. huCD3, human CD3; huCD45, human CD45.

These varying immune populations affected PDX growth, and mHM_TA_ tumors grew significantly slower than those on mHM [linear mixed-effects model, *P* = 0.007, when testing the β coefficients for the group indicator with tumor burden (i.e., the total volume of all tumors) as the outcome; Fig. 4A, and *P* = 0.003, using the sample model with tumor volume as the outcome; Fig. 4B]. Using Kaplan–Meier survival analysis, mHM_TA_ had longer PFS, when progression was defined as a tumor burden >180 mm^3^, with an increase of 20% over the baseline of 150 mm^3^ (or 50 mm^3^ volume per tumor) compared with mHM, with median time to progression of 57 days (95% confidence interval, 50–59) in mHM versus 62 days (95% confidence interval, 59–67) in mHM_TA_ (*P* < 0.001; Fig. 4C). This analysis mimics clinical progression criteria. In analyzing viable tumor burden (25), Ki67 staining showed that tumors from mHM_TA_ consisted of only a thin outer shell of live/proliferating cells surrounding a necrotic center (Fig. 4D), whereas those from mHM had a depth of viable tissue that was more than twofold thicker (*P* = 0.048, two-group *t* test; Fig. 4E). The total and viable tumor volumes were both significantly smaller in mHM_TA_ (Fig. 4F and G; *P* = 0.019 and *P* = 0.034, respectively, two-group *t* test).

We examined intratumoral T-cell populations from mHM and mHM_TA_, first by cytometry and then by IHC. Although some melanoma tumors are profoundly resistant to T-cell infiltration (a condition referred to clinically as a “cold tumor”; ref. 26), we observed human CD45^+^ cells within both mHM and mHM_TA_ cohorts by cell cytometry (Fig. 4H). Among these, intratumoral T cells trended slightly higher in mHM_TA_ (Fig. 4I), and IHC analysis—a more reliable tool, when examining the hard-to-digest tumor capsule—of human CD45^+^ and CD3^+^ cells present in nonoverlapping ∼250 μm^2^ fields in the tumors, and their capsules (Fig. 4J) revealed larger T-cell populations in the non-necrotic regions of the mHM_TA_ tumors (Fig. 4K and L; *P* = 0.0204 and 0.0107, respectively, Wilcoxon rank-sum test). The linkage of lower tumor volume and cell viability to intratumoral T-cell infiltration is significant (27) given their robust association in current therapeutic practice with tumor-infiltrating lymphocytes in melanoma, lending value to the mHM_TA_ system.

### Intratumoral heterogeneity analysis of PDX tumor tissue identifies neoAgs selected against in mHM_TA_

**Figure 5.**
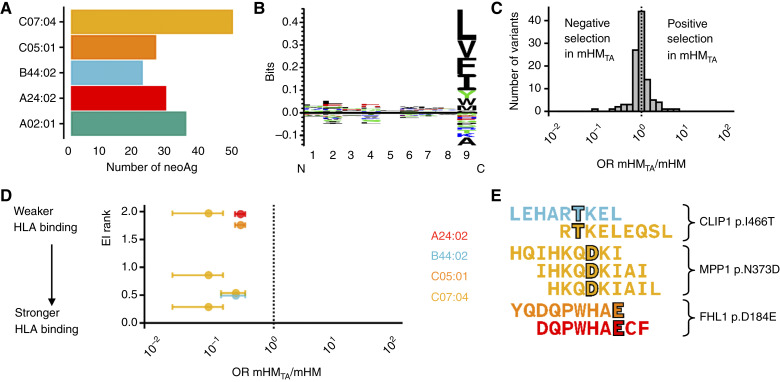
Changes in intratumoral heterogeneity in mHM_TA_ PDXs can be explained by neoAg selection. **A,** NeoAg distribution across HLA alleles (**B**) Kullback–Leibler logos show enrichment of hydrophobic amino acids at position 9 in the neoAg pool. The height of each one letter amino acid code is the absolute enrichment relative to unselected peptides. Values above the *x* axis are positively enriched whereas those below are depleted. **C,** Distribution of estimated changes in variant allele frequency between the mHM_TA_ and mHM models in all variants identified by exome sequencing. Values to the right of the dotted line represent variants that enriched, or underwent positive selection in mHM_TA_, whereas values to the left represent variants that underwent negative selection. **D,** Significant changes between mHM and mHM_TA_ variant allele frequencies are plotted against the predicted HLA binding affinity for nine-mer peptides derived from each variant. The points are colored by which class I HLA molecule the peptide was predicted to bind. All significantly altered variants were found to produce at least two neoAgs. Error bars represent SE. **E,** Single letter codes for each amino acid are shown with the mutant residue outlined in each. The majority of mutations occurred at TCR contact residue locations (positions 3–8).

To evaluate changes in intratumoral heterogeneity driven by varying immune permissiveness, we next employed high-depth exome sequencing of mHM_TA_, mHM, and originating patient tumor tissue. DNA samples were obtained to represent spatially distinct and heterogeneous cancer cell populations. We focused our analysis on somatic nonsynonymous protein-coding variants that were detectable in the patient sample. First, we used NetMHC to predict HLA binding for nine-mer peptides encompassing the mutated protein sequence. We defined putative neoAg as peptides with an eluted ligand rank of less than 2%. The distribution of neoAg binding HLA alleles revealed a high proportion of HLA-C*07:04 neoAg, followed by HLA-A*02:01 neoAg (Fig. 5A). Kullback–Leibler logos were created for the neoAg pool which show a strong enrichment of nonpolar amino acids leucine, valine, phenylalanine, and isoleucine at the c-terminal anchor residue (position 9; Fig. 5B).

Next, we analyzed differences in intratumoral heterogeneity that occurred in the mHM_TA_ compared with mHM. For each variant, regardless of predicted antigenicity, we calculated the variant allele frequency as the fraction of reads supporting the variant allele to the total read depth at that locus. Using binomial regression, we estimated the OR of encountering a variant read in mHM_TA_ relative to mHM tumors (Fig. 5C). ANOVA analysis using an overall *F* test, followed by FDR adjustment for multiple tests, identified three variants, *CLIP1* p.1466T, *MPP1* p.N373D, and *FHL1* p.D184E that were significantly different between the mHM and mHM_TA_ experimental arms. All three variants were less abundant in mHM_TA_ tumors (OR ± SE = 0.23 ± 0.10, 0.08 ± 0.06, and 0.27 ± 0.05, respectively) and gave rise to a total of seven neoAgs (Fig. 5D). Although the total neoAg pool illustrates the biological importance of a hydrophobic amino acid at the c-terminal anchor residue, the variants that were selected against in the mHM_TA_ were more likely to contain mutations in the TCR contact residues at positions 3 to 8. Two neoAgs, YQDQPWHAE and RTKELEQSL, were found to have anchor residue mutations at the c- and n-terminus ends, respectively (Fig. 5E).

## Discussion

Unless the T-cell lineage within an HM PDX model is representative of that found in the corresponding patient, the basis of immune-driven anticancer effect cannot be effectively studied. We sought to generate a model that can circumvent the challenge of obtaining autologous HSPCs to humanize mice by deriving thymic precursors from autologous iPSCs to the patient tumor to enable matched T-cell development and education, and we conducted a study to characterize autologous sTO capabilities both *in vitro* and *in vivo*.

The majority of previously described HM PDX models are engrafted with HSPCs from cord blood, and their resulting immune cells are allogeneic to subsequently implanted patient tumors (28–31). HLA-matching and transgenic models attempt to minimize tissue mismatches, but matching is often restricted, if done at all, to only the *HLA-A* and *HLA-B* alleles (32, 33). Several models have been described in which donor or patient PBMCs are used to humanize mice and tumors are subsequently implanted (34–36). Although this approach can be successful, these mice rapidly succumb to GVHD, limiting their usefulness in extended tumor treatment studies. Finally, HM have been generated from the surgically harvested bone marrow of patients with cancer although this type of model requires intrusive surgery and is limited to patients in whom this is done as part of their regular care (37). We recently described an autologous HM PDX model generated from HSPCs isolated after G-CSF mobilization in patients who subsequently underwent biopsies to establish PDX tissues (2). Such a model serves as a reproducible platform in which patient immune cells and tumors can coexist and already contains two of the three components necessary to recreate a clinical cancer tumor microenvrionrment.

Although several HM PDX models exist in which T-cell education occurs in a murine thymus or in implanted fetal thymic tissue, these T cells are exclusively educated in a xenogeneic or allogeneic environment (9, 38). In an HM model that pairs surgically excised neonatal thymus with HSPCs isolated from UCB (11), the T cells educated in this context will be allogeneic to implanted PDX tissue. It has not been previously feasible to procure patient thymic tissue to incorporate into HM PDX models. Although there have been recent reports of thymic organoids produced from iPSCs (39, 40), we here demonstrate that PBMCs from patients with cancer can be reprogrammed to iPSCs *in vitro*. Patient iPSCs can then be differentiated into TECs in the form of sTOs (13), which can be implanted into an HM, in which they will direct T-cell education in an environment autologous to that of the patient. Such a model represents a significant step in the *ex vivo*/*in vivo* production of patient-derived thymic tissue in culture. Using this sTO culture system, we have generated functional human patient-specific iPSC-derived TECs *in vitro* and *in vivo*. TECs within sTOs express genes critical for negative selection and facilitate the development of proT cells and DP T cells from allogenic and *HLA-A*–matched human cord blood CD34^+^ cells. Although the success rate of sTO engraftment could be further optimized for widespread utilization, our work provides proof of principle that PBMCs can be used to generate autologous thymic tissue matched to patient tumor tissue, a system with significant potential.

For these experiments, we used a cohort of HM engrafted with *HLA-A*–matched cord blood–derived HSPCs, and even in this semi-matched environment, we were able to demonstrate that T cells educated by implanted sTOs ultimately reduced the growth rate of autologous tumors. The sTO tissue produced DP, CD4^+^, and CD8^+^ T cells could also be detected in the spleen. This more comprehensive sTO-mediated T-cell education was also apparent in the splenocyte cytometric analysis, in which the mHM_TA_ had greater populations of PD-1–/HLA-DR+ T cells than the mHM controls, indicative of previously activated cells not subject to subsequent PD-1–mediated exhaustion.

Most significantly, from a clinical perspective, the tumor-invading T-cells produced in the mHM_TA_ model show an increased capacity to reduce the rate of tumor growth early in the study. Indeed, their effect would be most pronounced in tumors shortly after implantation, in which even a limited number of active neoAg-recognizing T cells would have a prominent effect on tumor cell proliferation. Those T cells remaining at the time of tumor tissue collection would be concentrated near the proliferating edge of the tumors, as observed in the IHC of the collected tissues. A comparison of these proliferating outer layers of the mHM and mHM_TA_ tumors provides additional evidence of the activity of these T cells. In the mHM, this proliferating edge makes up approximately 35% of the total tumor volume, whereas in the mHM_TA_, living cells compose only 25% of the total tumor volume, the remainder consisting of a necrotic central core.

Increased T-cell activity in the mHM_TA_ can also be inferred from a genomic analysis of the tumors. Intratumoral heterogeneity is well represented in exome sequencing data and can be used to monitor tumor dynamics in response to treatment (41, 42). Exome profiling of the PDX tumors showed a lower abundance of subclonal mutations in the mHM_TA_ model, which is consistent with effective immunoediting in the less permissive, CD8 tumor microenvritonmnt-enriched mHM_TA_ system. Furthermore, by assessing genomic rather than transcriptomic changes, we posit that the changes reported are a result of stable clonal selection and not transient changes in gene expression. We identified seven putative neoAgs that were significantly altered between the mHM and mHM_TA_ tumors. None of the neoAg that were significantly altered were HLA-A*02:01 binders, which was the only allele that was matched between HSPCs and the patient. Although further studies are needed to corroborate this finding, it is possible that neoAgs are presented via tolerogenic A02:01+ dendritic cells in the sTO. Although this mechanism of peripheral tolerance has been well described in mice (43), little is known about its role in cancer immunotherapy. Using the mHM_TA_ model with intentionally HLA-mismatched HSPCs may help elucidate the role of tolerogenic dendritic cells in the evolution of cancer. Another application of this model relates to cancer vaccine development and neoAg target identification; although HLA binding predictions are widely used to predict neoAgs from variant calls, there are well-documented limitations to this approach (44). It is possible that variants identified in this report as having an OR < 1 may be *bona fide* neoAgs despite not being predicted to bind HLA class I molecules. Using advanced HM models in future research may address these complex issues preclinically.

We show that patient-autologous T-cell education in an HM PDX model is possible and produces active T cells representative of those found in the patient. We also demonstrate how these T cells can slow tumor growth. This work bridges the gap between the previously described autologous HM model and a mouse model employing human thymic tissue. A merging of these technologies will accelerate the development of a mouse model in which immune cells, thymic tissue, and tumor tissue are all procured from a single patient.

## Supplementary Material

Supplementary Methods Supplemental Methods

Supplemental Figure 1 Supplemental Figure 1

Supplemental Figure 2 Supplemental Figure 2

Supplemental Figure 3 Supplemental Figure 3

Supplemental Figure 4 Supplemental Figure 4

Supplemental Figure 5 Supplemental Figure 5

Supplemental Figure 6 Supplemental Figure 6

Supplemental Figure 7 Supplemental Figure 7

Supplemental Figure 8 Supplemental Figure 8

Supplemental Table 1 Supplemental Table 1

## Data Availability

Data and materials were generated by the authors and are available upon request from the corresponding author. All data will be shared per the University of Colorado’s Office for Technology Transfer policies and Institutional Review Board. All code used for analysis is available at github.com/jimeno-lab. This study is pending registration with Database of Genotypes and Phenotypes.
