# Supplementary material for: Autologous Thymic Organoids Support Functional T-cell Education and Enhance Antitumor Immunity in Humanized Mice with Melanoma Xenografts
Source: Cancer Res Commun. 2025 Nov 24;5(11):2053–65. doi: 10.1158/2767-9764.CRC-25-0357 (PMC12641387; doi:10.1158/2767-9764.CRC-25-0357)
Supplement: Supplementary Methods — Supplemental Methods [file crc-25-0357_supplementary_methods_suppsm.docx]

**SUPPLEMENTAL MATERIALS AND METHODS**

**Glossary of abbreviations**

DP T cells – Double-positive (CD4+/CD8+) T cells

HSPC – human stem and progenitor cells

iPCSs – induced pluripotent stem cells

mHM – mismatched humanized mouse, produced by the engraftment of *HLA-A* matched HSPCs

mHM_TA_ – mismatched humanized mouse in which thymic tissue autologous to the patient tumor has been implanted in the kidney capsule

PBMCs – peripheral blood mononuclear cells

PDX – patient-derived xenograft

sTO – stem cell-derived thymic organoids

TEPs – thymic epithelial progenitors

TECs – thymic epithelial cells

UCB – umbilical cord blood

**Animal care and PDX generation**

Previously cryopreserved patient tumor samples were transported in fresh RPMI, supplemented with 10%FBS and pen/strep, cut into 3mm cubes, then immersed in Matrigel and implanted subcutaneously into a small pocket beneath the skin on both flanks and a shoulder of isoflurane-anesthetized NSG mice. Animals were monitored during tumor growth, and once the PDX tissue had grown sufficiently they were removed to allow passage onto the mHM/mHM_TA_ cohorts, it was removed, and this procedure repeated on the study animals. HM from UCB120 received F7 tumors, while UCB122 and UCB139 HM were implanted with F6 tissue. Tumors were measured twice-weekly using digital calipers, and the tumor volumes were calculated using the formula (WxWx L)/2. When any tumor’s volume became greater than 1500mm^3^, or the total tumor burden of the mouse exceeded 3000mm^3^, the mouse was euthanized and the tumors removed for analysis.

**sTO analysis**

For flow cytometry analysis, sTOs were harvested by adding 0.5mL of FACS buffer (0.1% BSA and 2mM EDTA in PBS) to each membrane, mechanically dissociating the sTOs, and passing the sTO suspensions through a 40µm FACS filter into a 5mL round bottom polystyrene FACS tube. For qPCR analysis, sTOs were harvested with 0.5mL FACS buffer, transferred to a 1.5mL tube, and pelleted by centrifugation. The cell pellet was resuspended in 350µL of Qiagen buffer RLT lysis buffer (Qiagen, Germantown, MD, Cat#79216), stored at -80°C, and RNA was isolated using a RNeasy Mini Kit (Qiagen, Cat#74104) per manufacture instructions. To prepare sTOs for Immunofluorescence (IF), 4% PFA was added, and sTOs were fixed for 15 minutes at room temperature. The sTOs were washed three times with PBS, which was then replaced with 30% sucrose, and stored overnight at 4°C. sTOs were then embedded in OCT Compound (Leica Biosystems, Wetzlar, Germany, Cat#39475237) in a sectioning mold, snap frozen, and stored at -80°C.

**sTO characterization**

Real-time quantitative PCR was performed on a CFX96 Touch Real-Time PCR Detection System (Bio-Rad Laboratories, RRID:SCR_018064) using human-specific Taqman probes (Bio-Rad Laboratories or ThermoFisher Scientific, Waltham, MA) or human-specific primers listed below. Samples were normalized to endogenous control gene *ACTB* and plotted relative to undifferentiated iPSCs.

| Probe Target | Supplier: Assay ID | Primer Name | Sequence |
| --- | --- | --- | --- |
| ACTB | ThermoFisher: Hs01060665_g1 | ACTB For  ACTB Rev | CATGTACGTTGCTATCCAGGC  CTCCTTAATGTCACGCACGAT |
| ACTB | ThermoFisher: Hs99999903_m1 | Pro-insulin For  Pro-insulin Rev | GCAGCCTTTGTGAACCAACAC  CCCCGCACACTAGGTAGAGA |
| AIRE | Bio-Rad: qHsaCIP0029272 | Islet Antigen 2 For  Islet Antigen 2 Rev | CGGGACACATGATTCTGGCAT  CTGCTTGGTAGGCACAGAGG |
| DLL4 | Bio-Rad: qHsaCEP0051500 | GAD1 For  GAD1 Rev | GCGGACCCCAATACCACTAAC  CACAAGGCGACTCTTCTCTTC |
| FOXN1 | ThermoFisher: Hs00919266_m1 | MBP For  MBP Rev | GGCCGGACCCAAGATGAAAA  CCCCAGCTAAATCTGCTCAGG |
| HLA-DRA | Bio-Rad: qHsaCEP0040019 | TG For  TG Rev | AGACACCTCCTACCTCCCTCA  TCCTTGGACATCGCTTTGGC |
| KRT5 | Bio-Rad: qHsaCEP0055058 |  | |
| KRT8 | Bio-Rad: qHsaCEP0041467 |  |  |

**Mouse thymectomies**

Prior to humanization, cohorts of mice were thymectomized. Surgery was slightly modified from a previously published procedure (1). Briefly, six-week NSG mice were induced with ketamine/xylazine and intubated using a RoVent small animal ventilator (Kent Scientific, Torrington, CT). The ventilator was set to maintain a small positive expiratory end pressure (PEEP) to prevent pneumothorax once the chest wall was opened. Once stably intubated, mice were given 0.05mL buprenorphine SR (Ethiqa; Fedelis Animal Health, North Burnswick, NJ). A surgical anesthesia plane was achieved and maintained using isoflurane. Mice were positioned dorsally on an infrared heating pad with homeostatic control. A longitudinal incision measuring approximately 1cm and centered at the sternal notch was made, exposing the submandibular salivary gland. The salivary gland was moved rostral and supported by a moistened gauze sponge to prevent desiccation. An English nail anvil was used to divide the sternum from the sternal notch to the second rib. The strap muscles were then bluntly divided longitudinally to expose the mediastinum. The thymus was grasped with corneal forceps and removed with micro scissors. The specimen was fixed in formalin and assessed by IHC. The chest wall was closed with 6-0 polyglactin to reapproximate the sternum, followed by 6-0 silk suture for the skin. Animals were weaned from the ventilator over the course of 30 minutes, or until spontaneous respirations occurred. If no spontaneous respirations occurred, Dopram (Baxter Pharmaceuticals, Deerfield, IL) was administered. Animals were then allowed to recover on a warming blanket then monitored daily until complete recovery.

**HSPC purification, expansion, and engraftment**

HSPCs were purified from either cord or patient blood by CD34+ positive cell selection (Stemcell Technologies, Cat#14756), suspended in serum-free expansion medium (Stemcell Technologies, Cat#09650), and cultured at 37°C, 5% CO_2_ for 5-8 days. Cells were characterized by cytometry, using CD34, CD45, CD73, and CD166 antibodies (Biolegend, Cat#343608, RRID:AB_2228972; 304039, RRID:AB_2562057; 344006, RRID:AB_1877157; 343904, RRID:AB_2289302) at a 1:10 concentration. NSG (Jackson Laboratories, Bar Harbor, ME; Cat#005557, RRID:IMSR_JAX:005557) mice were primed for engraftment by 1.5Gy whole-body irradiation. After a recovery of 4-6 hours, the mice were each injected with 400,000 expanded CD34+ cells, suspended in 0.2mL sterile PBS. When present, MSC-like cells were added to comprise 5% of the total injected cells. The mice were bled via the tail vein every two weeks to assess HSPC engraftment. Their peripheral blood was analyzed by flow cytometry, using human CD3, CD11b or CD14, CD19 and/or CD45 (Biolegend; Cat#300312, RRID:AB_314048; 301310, RRID:AB_314162; 367118, RRID:AB_2566792; 392504, RRID:AB_2728416; 304039, RRID:AB_2562057) antibodies all at 1:10.

**IHC analysis**

Primary antibodies and dilutions: CD45 (Dako; Cat#M0701, RRID:AB_2314143), 1:100; CD3 (Abcam; San Francisco, CA; Cat#ab5690, RRID:AB_305055), 1:500; hKRT5 (ThermoFisher; Cat#PA1-37974, RRID:AB_2134167), 1:200; hKRT8 (Abcam; Cat#ab15465, RRID:AB_301875), 1:500; Ki67 (ThermoFisher; Cat#RM-9106-S, RRID:AB_2341197), 1:100. Staining was developed using the following conditions: EnVision + Dual Link System HRP (Dako; Cat#K4061) for 30 minutes and substrate-chromogen (DAB+) Solution (Dako; Cat#K3468) for 5 minutes. Slides were then counterstained with Automated Hematoxylin (Dako; Cat#S3301) for 10 minutes. For double staining, Dual Endogenous Enzyme Block (Dako; Cat# S2003) for 10 minutes, Protein-free Block (Dako; Cat # X0909) for 20 minutes before the first primary antibody for 1 hour, followed by EnVision HRP for 30 minutes and substrate-chromogen (DAB+) for 5 mins. Slides were washed with buffer and blocker before the second primary antibody was used. Staining was developed using MACH2 AP Polymer (BioCare; cat#RALP525) for 30 mins, followed by Vulcan Fast Red (BioCare; #FR805), and then counterstained with hematoxylin (Dako; Cat#S3301) for 10 mins.

**Whole exome sequencing**

Samples of tumor tissue were flash-frozen in liquid nitrogen at the time of tissue collection, and stored at -80°C. For genomic DNA extraction, the frozen tissue was ground with a mortar and pestle under liquid nitrogen. The pulverized tissue was digested with Proteinase K for 1 hour at 56°C and DNA was isolated using phenol-chloroform and ethanol precipitation (2) (3). CUHM009 DNA isolates were overtly contaminated with melanin, a PCR inhibitor. To clean up the CUHM009 DNA samples and avoid potential confounding variables we opted to treat all DNA samples with the OneStep PCR Inhibitor Removal Kit (Zymo Research, Irvine, CA; Cat#D6030).

**Genomic sequencing and data analysis**

We used a combination of the HLA genotype calls from BWA kit and high-resolution HLA typing results provided by the Barbara Davis Center for Childhood Diabetes HLA Laboratory to obtain HLA types. Following alignment, PCR and optical sequencing duplicates were removed using Picard MarkDuplicates (RRID:SCR_006525) (4). Base quality scores were recalibrated using the BaseQualityRecalibrator tool and ApplyBQSR. Somatic variants were called using Mutect2 in multi-sample tumor-normal mode with each patient and the PDXs derived from their tumor representing one cohort. Genomic DNA from PBMCs was used for the normal sample in each case. Variants were annotated using GATK Funcotator and filtered for high confidence calls (5). We next identified somatic non-synonymous variants and inferred the mutant amino acid sequence resulting from the mutation. We then used a sliding window approach to produce *in-silico* “digested” nine amino acid length peptides that incorporated the variant region. Using NetMHCpan-4.1 (6), we predicted the variant peptides’ affinity for each class-I HLA molecule within the patient’s HLA genotype.

**Statistical Tests**

For Figure 1 Panels B and C, relative quantification was performed using TBP as a housekeeping gene. The ΔΔCt value for each target was tested for significant differences using analysis of variance with categorical independent variable levels iPSC, sTOC-d0, sTOC-wk3, sTOC-wk6. Statistically significant comparisons were identified using Tukey’s Honest Significant Differences. For Figure 3 Panels B, C, and E, the percentage of target cells identified using flow cytometry was subject to a Wilcoxon Rank Sum test between the mHM and mHM_TA_ groups. For Fig 3 Panel F, a negative binomial generalized linear model was fit to the data. The count of IFN-ɣ positive cells was weighted by the tissue area examined (mm^2^) then fit to the experimental arm (NSG, mHM, mHM_TA_). The ‘emmeans’ package was then used to test for pairwise differences with Tukey’s correction for multiple tests (<https://rvlenth.github.io/emmeans/>). For Figure 4 Panels A and B, the ‘lmer’ package was used to fit a mixed log-linear effects model with the log of tumor burden as the response variable and the days post-implantation, arm, and interaction of the arm with days post-implantation as the explanatory variables (7). The random effects were specified as the cohort and, nested within the cohort, the mouse identifier. Type II Wald Chi Squared tests were performed on each explanatory variable to determine statistical significance. For Figure 4 Panel C, Kaplan-Meier survival curves were created using the ‘survival’ package where the endpoint was tumor progression as defined as a 20% increase in overall tumor burden. The ‘survminer’ package was used to assess the difference in progression free survival between mHM and mHM_TA_. For Figure 4 Panels H and I, differences between mHM and mHM_TA_ were assessed using a Wilcox Rank Sum Test.

**SUPPLEMENTAL REFERENCES**

1. Khosravi-Maharlooei M, Hoelzl M, Li HW, Madley RC, Waffarn EE, Danzl NM*, et al.* Rapid thymectomy of NSG mice to analyze the role of native and grafted thymi in humanized mice. Eur J Immunol **2020**;50:138-41

2. Green MR, Sambrook J. Precipitation of DNA with Ethanol. Cold Spring Harb Protoc **2016**;2016

3. Green MR, Sambrook J. Isolation of High-Molecular-Weight DNA Using Organic Solvents. Cold Spring Harb Protoc **2017**;2017:pdb prot093450

4. Meraz IM, Majidi M, Meng F, Shao R, Ha MJ, Neri S*, et al.* An Improved Patient-Derived Xenograft Humanized Mouse Model for Evaluation of Lung Cancer Immune Responses. Cancer Immunol Res **2019**;7:1267-79

5. McKenna A, Hanna M, Banks E, Sivachenko A, Cibulskis K, Kernytsky A*, et al.* The Genome Analysis Toolkit: a MapReduce framework for analyzing next-generation DNA sequencing data. Genome Res **2010**;20:1297-303

6. Reynisson B, Alvarez B, Paul S, Peters B, Nielsen M. NetMHCpan-4.1 and NetMHCIIpan-4.0: improved predictions of MHC antigen presentation by concurrent motif deconvolution and integration of MS MHC eluted ligand data. Nucleic Acids Res **2020**;48:W449-W54

7. Bates D, Mächler M, Bolker BM, Walker SC. Fitting Linear Mixed-Effects Models Using lme4. J Stat Softw **2015**;67:1-48
