# Supplementary material for: Autologous Thymic Organoids Support Functional T-cell Education and Enhance Antitumor Immunity in Humanized Mice with Melanoma Xenografts
Source: Cancer Res Commun. 2025 Nov 24;5(11):2053–65. doi: 10.1158/2767-9764.CRC-25-0357 (PMC12641387; doi:10.1158/2767-9764.CRC-25-0357)
Supplement: Supplemental Figure 1 [file crc-25-0357_supplemental_figure_1_suppsf1.docx]

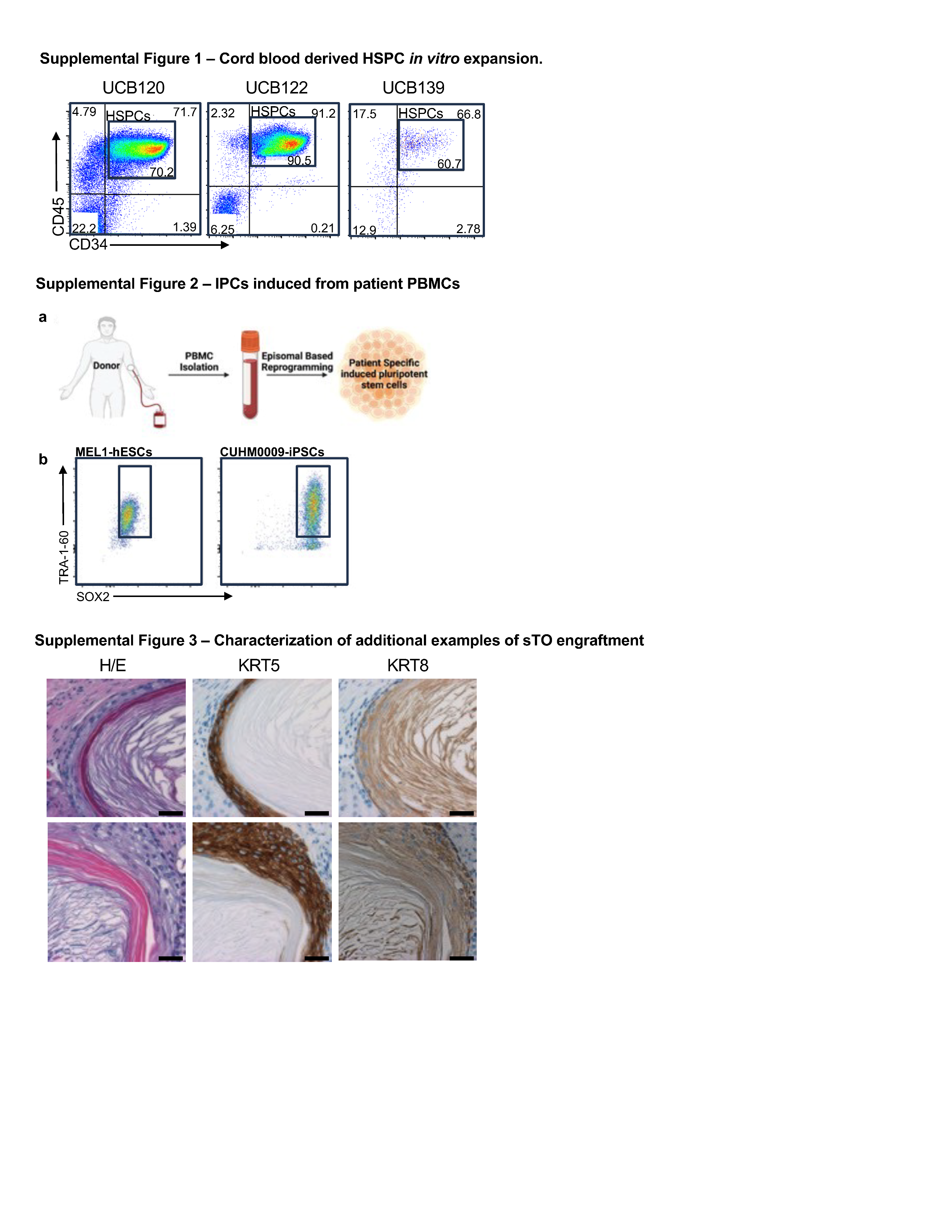


**Supplemental Figure 1. Cord blood HSPC *in vitro* expansion.** After expansion, UCB120 HSPCs increased from an initial population of 790,800 to a final population of over 30 million, UCB122 HSPCs increased from 646,000 to over 43 million, and UCB139 HSPCs expanded from 62,760 to approximately 33.5 million, enabling the generation of HM cohorts and *HLA-A* matched sTOs from each cord.
