# Supplementary material for: Autologous Thymic Organoids Support Functional T-cell Education and Enhance Antitumor Immunity in Humanized Mice with Melanoma Xenografts
Source: Cancer Res Commun. 2025 Nov 24;5(11):2053–65. doi: 10.1158/2767-9764.CRC-25-0357 (PMC12641387; doi:10.1158/2767-9764.CRC-25-0357)
Supplement: Supplemental Figure 2 [file crc-25-0357_supplemental_figure_2_suppsf2.docx]

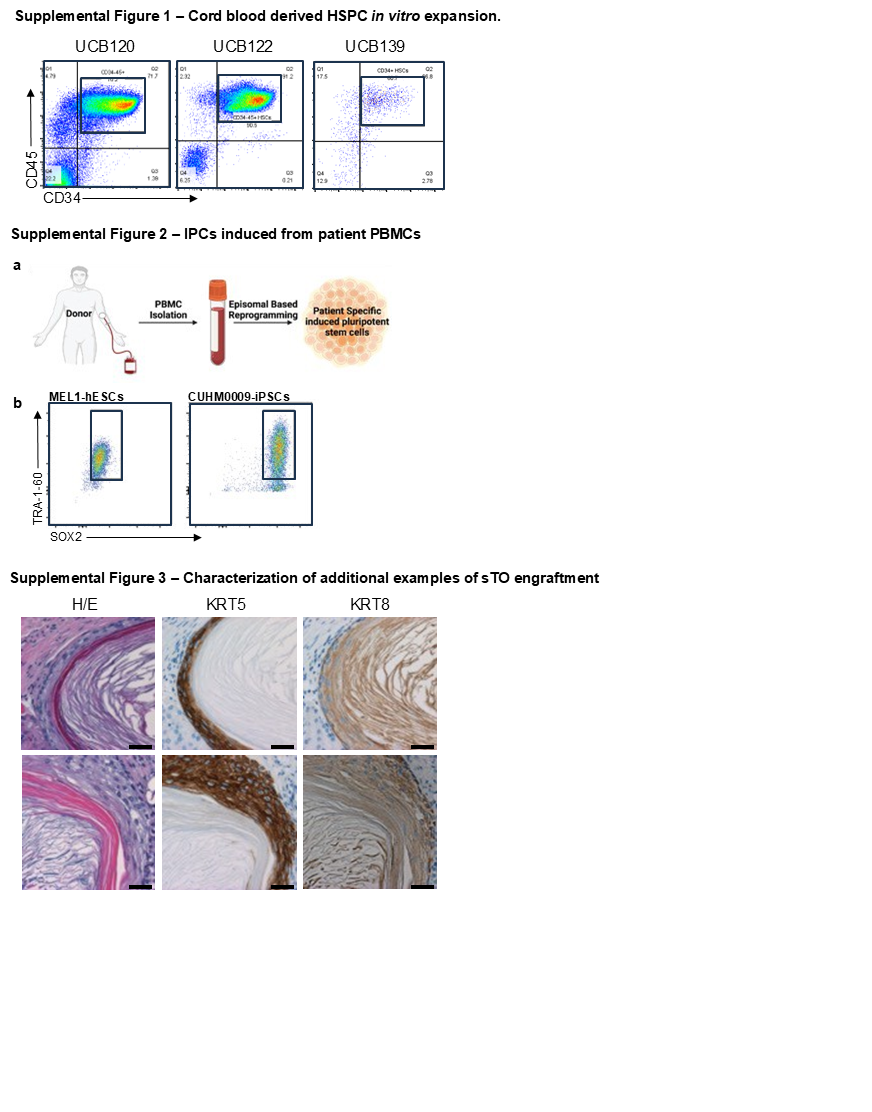


**Supplemental Figure 2. iPSCs induction from patient PBMCs. (a)** Overview of iPSC generation. Patient blood is drawn and the PBMCs isolated and reprogrammed into iPSCs. **(b)** The TRA-1-60 and SOX2 expression of the CUHM009 iPSCs was compared to that of the previously characterized Mel1-hESC cell line to verify successful reprogramming.
