# Supplementary material for: Autologous Thymic Organoids Support Functional T-cell Education and Enhance Antitumor Immunity in Humanized Mice with Melanoma Xenografts
Source: Cancer Res Commun. 2025 Nov 24;5(11):2053–65. doi: 10.1158/2767-9764.CRC-25-0357 (PMC12641387; doi:10.1158/2767-9764.CRC-25-0357)
Supplement: Supplemental Figure 3 [file crc-25-0357_supplemental_figure_3_suppsf3.docx]

**
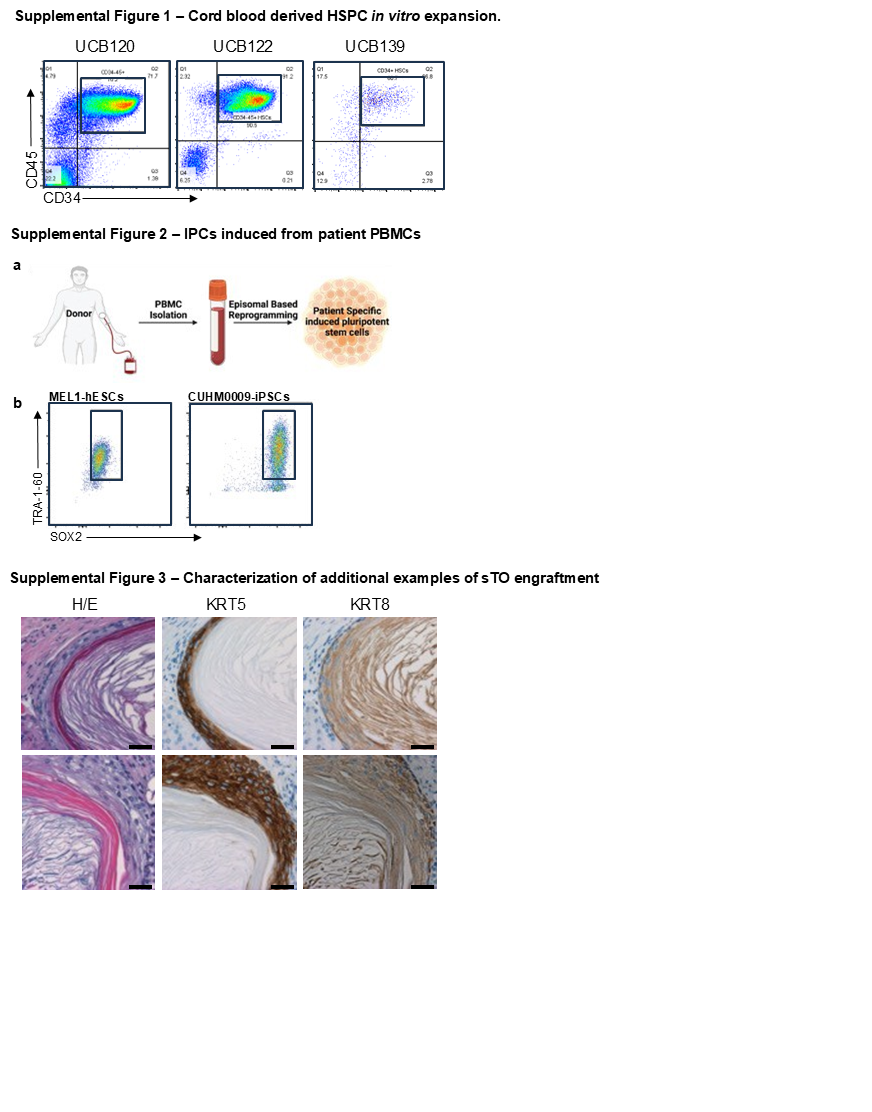
**

**Supplemental Figure 3**. **Characterization of additional examples of sTO engraftment.** IHC analysis of additional sTOs identified in mHM_TA_, showing the presence of human KRT5 and KRT8 proteins. Magnification is 20x; scale bar =50um.
