# Supplementary material for: Autologous Thymic Organoids Support Functional T-cell Education and Enhance Antitumor Immunity in Humanized Mice with Melanoma Xenografts
Source: Cancer Res Commun. 2025 Nov 24;5(11):2053–65. doi: 10.1158/2767-9764.CRC-25-0357 (PMC12641387; doi:10.1158/2767-9764.CRC-25-0357)
Supplement: Supplemental Figure 4 [file crc-25-0357_supplemental_figure_4_suppsf4.docx]

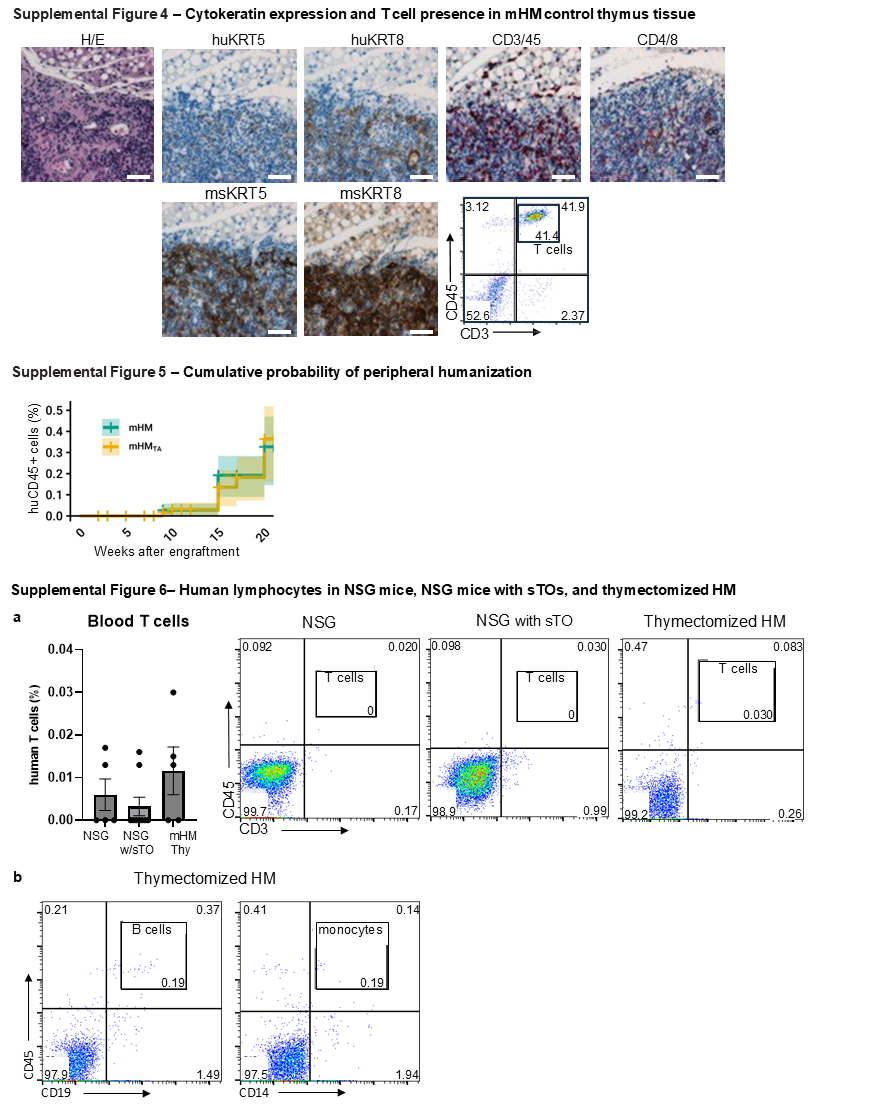


**Supplemental Figure 4. IHC of murine thymus expression.** IHC of representative murine thymus tissue from mHM showing expression of only murine cytokeratins (KRT5 and KRT8), although antibody cross-reactivity results in minimal human staining. CD3/45+ and CD4/8 DP T-cells can also be observed within this tissue. Magnification is 20x, scale bar = 50um. Cytometry of the murine thymus tissue also verifies the presence of CD3+ T-cells within this tissue.
