# Supplementary material for: Autologous Thymic Organoids Support Functional T-cell Education and Enhance Antitumor Immunity in Humanized Mice with Melanoma Xenografts
Source: Cancer Res Commun. 2025 Nov 24;5(11):2053–65. doi: 10.1158/2767-9764.CRC-25-0357 (PMC12641387; doi:10.1158/2767-9764.CRC-25-0357)
Supplement: Supplemental Figure 5 [file crc-25-0357_supplemental_figure_5_suppsf5.docx]

**
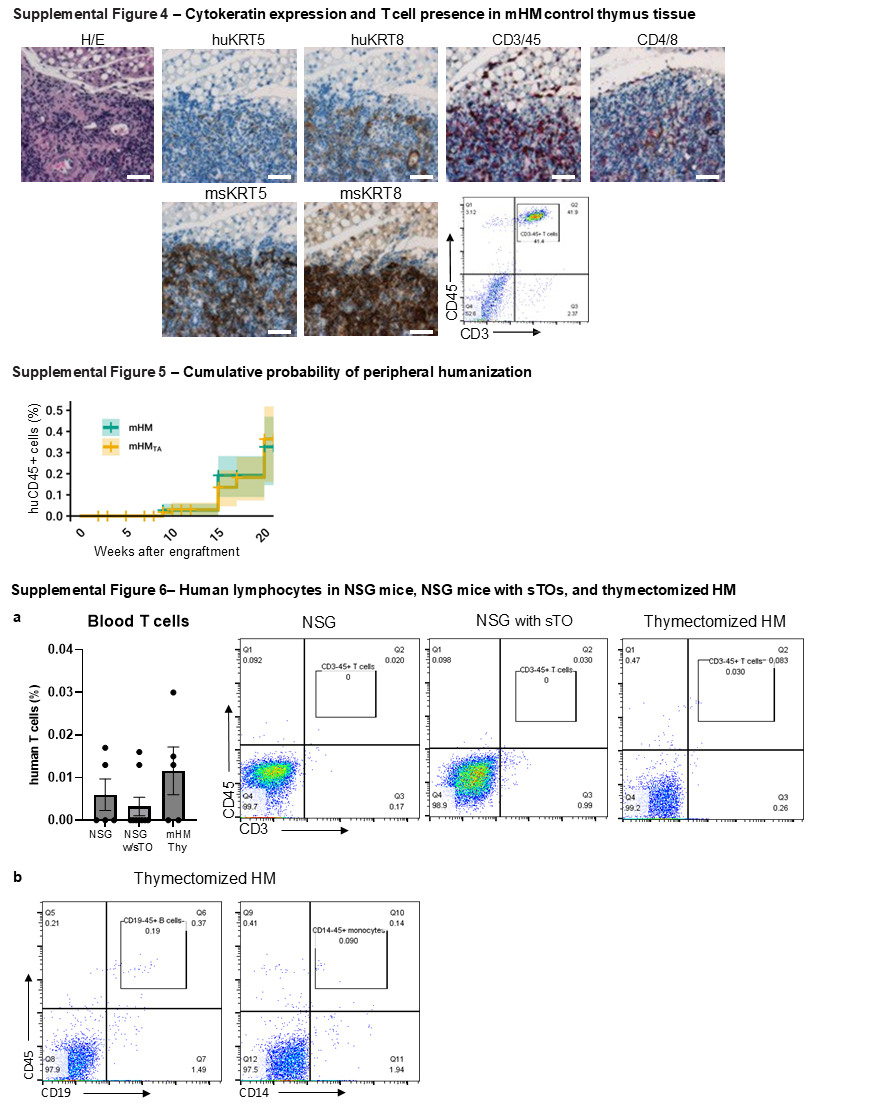
**

**Supplemental Figure 5. Cumulative probability of peripheral humanization.** Cumulative event survival plot representing the proportion of engrafted mice in each arm and the time to engraftment (defined as having >10% human CD45+ lymphocytes). Shading represents the 95% confidence interval. There was no significant difference between arms (p=0.92, cumulative hazard ratio).
