# Supplementary material for: Autologous Thymic Organoids Support Functional T-cell Education and Enhance Antitumor Immunity in Humanized Mice with Melanoma Xenografts
Source: Cancer Res Commun. 2025 Nov 24;5(11):2053–65. doi: 10.1158/2767-9764.CRC-25-0357 (PMC12641387; doi:10.1158/2767-9764.CRC-25-0357)
Supplement: Supplemental Figure 6 [file crc-25-0357_supplemental_figure_6_suppsf6.docx]

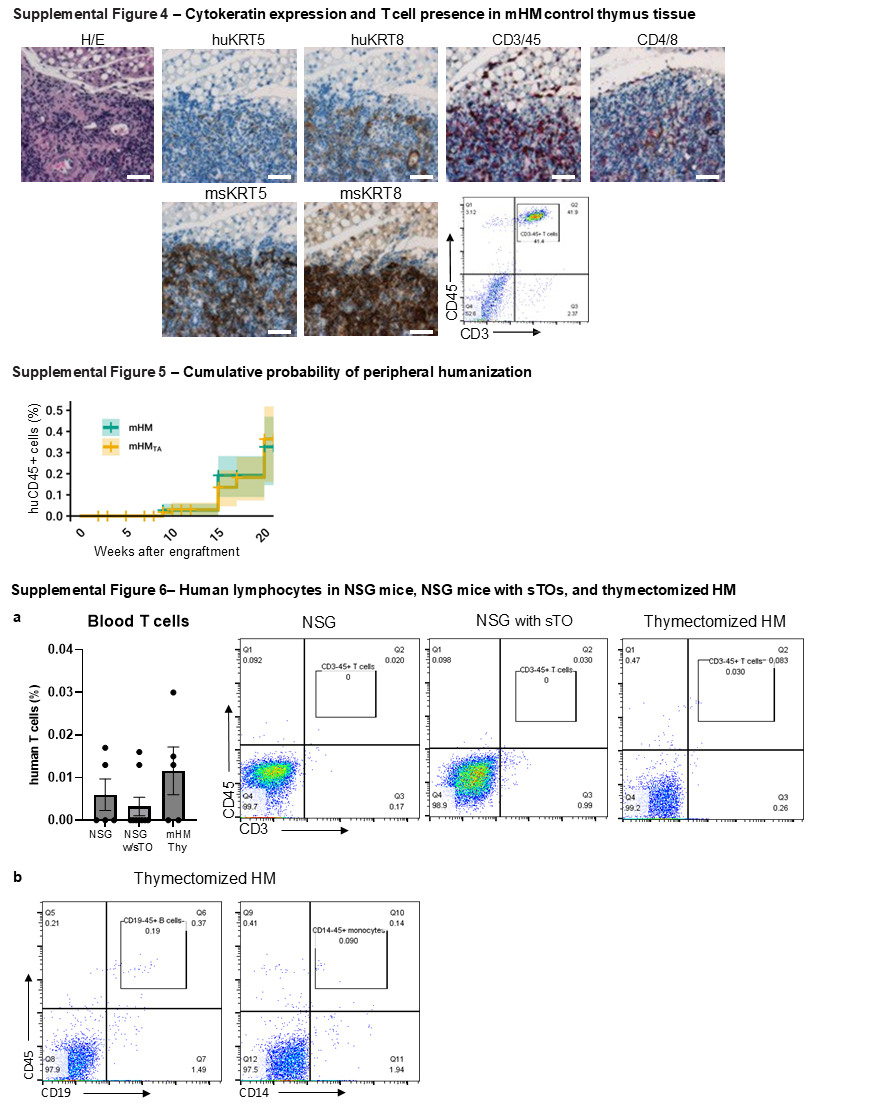


**Supplemental Figure 6. Human lymphocytes in NSG, NSGs with sTOs, and thymectomized HM. (a)** Comparison of T-cells found in the NSG, NSGs implanted with sTOs, and thymectomized mHM. Flow cytometry performed on the blood of representative mice indicates that no T-cells are present in NSG and NSG with sTOs. The existence of a very small population of putative T-cells in this thymectomized HM cannot be dismissed, but these cells are CD3-dim and are much less abundant than the other human cells in the mouse blood. **(b)** B-cells and monocytes identified in the blood of this mouse. In general, the percentage of these cells among all human CD45+ cells within the mouse blood was similar to that observed in mHM controls (B cells: 57.25% avg, 2.05% SEM; monocytes: 25.75% avg, 3.65% SEM).
