# Supplementary material for: Autologous Thymic Organoids Support Functional T-cell Education and Enhance Antitumor Immunity in Humanized Mice with Melanoma Xenografts
Source: Cancer Res Commun. 2025 Nov 24;5(11):2053–65. doi: 10.1158/2767-9764.CRC-25-0357 (PMC12641387; doi:10.1158/2767-9764.CRC-25-0357)
Supplement: Supplemental Figure 7 [file crc-25-0357_supplemental_figure_7_suppsf7.docx]

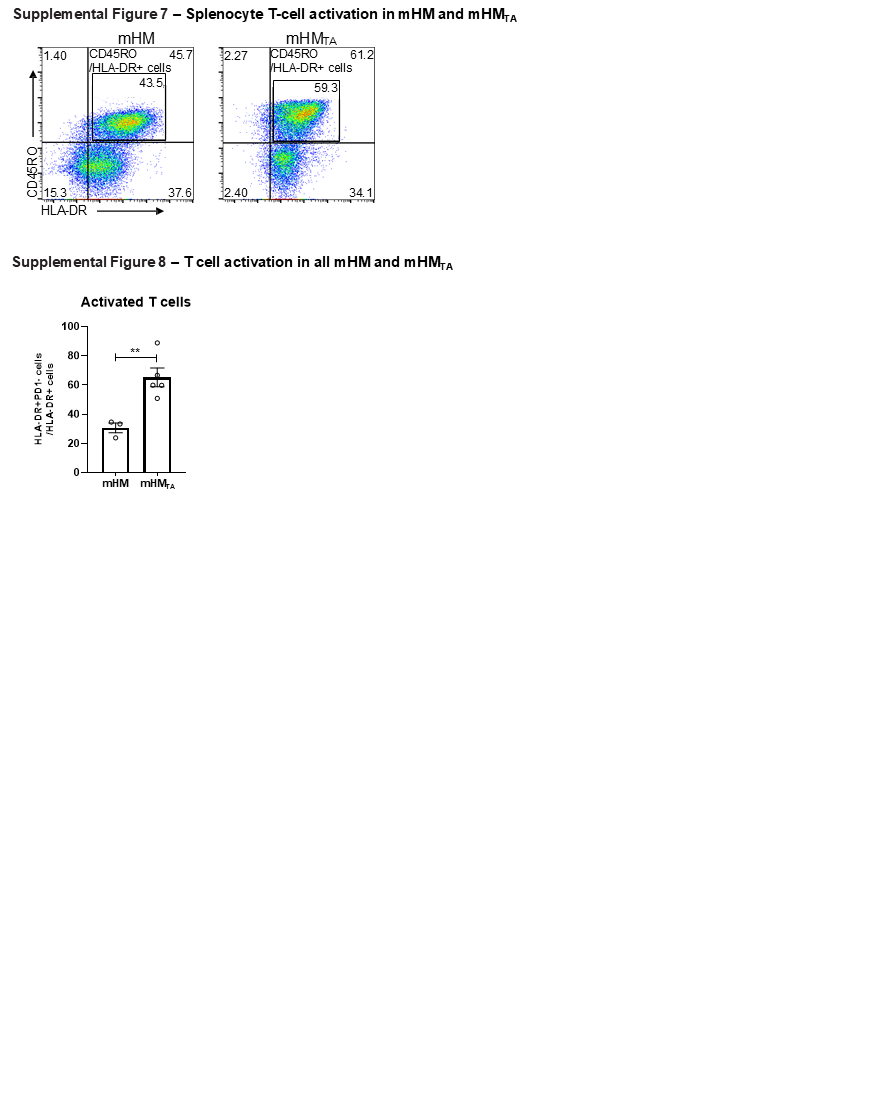


**Supplemental Figure 7. Splenocyte CD8+ T-cell activation in mHM and mHM_TA_.** Cytometric analysis of mHM and mHM_TA_ splenocytes indicates that a greater percentage are activated as assessed by CD45RO/HLA-DR+ cells in the mHM_TA_ spleen.
