# Supplementary material for: Autologous Thymic Organoids Support Functional T-cell Education and Enhance Antitumor Immunity in Humanized Mice with Melanoma Xenografts
Source: Cancer Res Commun. 2025 Nov 24;5(11):2053–65. doi: 10.1158/2767-9764.CRC-25-0357 (PMC12641387; doi:10.1158/2767-9764.CRC-25-0357)
Supplement: Supplemental Figure 8 [file crc-25-0357_supplemental_figure_8_suppsf8.docx]

**
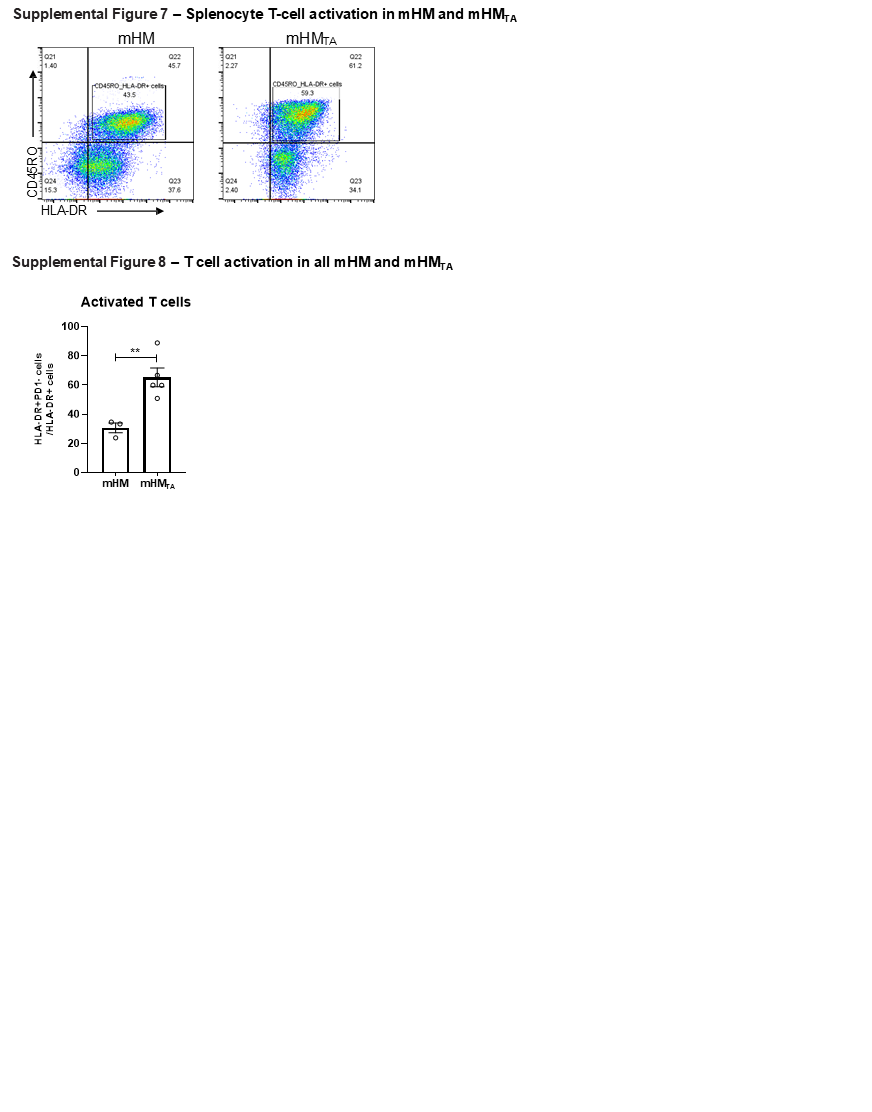
**

**Supplemental Figure 8. T-cell activation in all mHM and mHM_TA._** Comparison of the ratio of HLA-DR+PD1- T-cells to all HLA-DR+ T-cells in all mHM and mHM_TA_ (including those with <10% peripheral human cells) also shows an increasingly activated T-cell population in mHM_TA_ (p=0.0082, two-group t-test). P values: * ≤0.05, **≤0.01, ***≤0.001.
