# Supplementary material for: Autologous Thymic Organoids Support Functional T-cell Education and Enhance Antitumor Immunity in Humanized Mice with Melanoma Xenografts
Source: Cancer Res Commun. 2025 Nov 24;5(11):2053–65. doi: 10.1158/2767-9764.CRC-25-0357 (PMC12641387; doi:10.1158/2767-9764.CRC-25-0357)
Supplement: Supplemental Table 1 [file crc-25-0357_supplemental_table_1_suppst1.docx]

| **Locus** | **UCB120** | **UCB122** | **UCB139** | **CUHM009** |
| --- | --- | --- | --- | --- |
| **HLA-A** | ***02:01**/*25:01 | ***02:01**/*68:01 | ***02:01**/*11:01 | ***02:01**/*24:02 |
| **HLA-B** | *15:01/*51:01 | *39:06/*40:02 | *07:02/35:41 | *44:02/*44:02 |
| **HLA-DQA-1** | *01:03/*01:03 | *03:01/*05:03 | *01:01/*01:02 | *01:02/*03:03 |
| **HLA-DQB-1** | *06:03/*06:03 | *03:02/*03:01 | *05:01/*06:02 | *03:01/*06:04 |
| **HLA-DRB-1** | *13:01/*13:01 | *04:07/*14:06 | *01:03/*15:01 | *04:01/*13:02 |

**Supplemental Table 1: High Resolution HLA Typing Results**
